# P-305. No Clinically Significant Drug–Drug Interactions with Co-administration of Feminizing or Masculinizing Hormone Therapy and Twice-yearly Lenacapavir PrEP in Gender-Diverse Persons

**DOI:** 10.1093/ofid/ofaf695.525

**Published:** 2026-01-11

**Authors:** Jill Blumenthal, Maribel Acevedo-Quinones, Nittaya Phanuphak, Juan Carlos Hinojosa, Javier R Lama, Michelle S Cespedes, Nkosiphile Ndlovu, Carlos Brites, Sarah B Puryear, Lillian B Brown, Christoph C Carter, Priyanka Arora, Marjorie Z Imperial, Kim Etchevers, Renu Singh, Pamela Wong, Olivia T Van Gerwen

**Affiliations:** University of California San Diego, San Diego, CA; Centro Ararat, San Juan, Not Applicable, Puerto Rico; Institute of HIV Research and Innovation – Pribta Tangerine Clinic, Bangkok, Krung Thep, Thailand; Asociación Civil Selva Amazónica, Iquitos, Loreto, Peru; Asociación Civil Impacta Salud y Educacion, Lima, Lima, Peru; Icahn School of Medicine at Mount Sinai, New York, New York; Wits RHI, Faculty of Health Sciences, School of Public Health, University of the Witwatersrand, Johannesburg, Gauteng, South Africa; Complexo Hospitalar Universitário Professor Edgard Santos, Salvador, Bahia, Brazil; Gilead Sciences, Inc., Foster City, California; Gilead Sciences, Inc., Foster City, California; Gilead Sciences, Inc., Foster City, California; Gilead Sciences, Inc., Foster City, California; Gilead Sciences, Inc., Foster City, California; Gilead Sciences, Inc., Foster City, California; Gilead Sciences, Inc., Foster City, California; Gilead Sciences, Inc., Foster City, California; University of Alabama at Birmingham, Birmingham, Alabama

## Abstract

**Background:**

Transgender and gender-diverse persons are priority groups in HIV prevention; however, concerns over potential drug–drug interactions between gender-affirming hormone therapy (GAHT) and pre-exposure prophylaxis (PrEP) may hinder PrEP uptake. We report the pharmacokinetic (PK) effects of co-administration of masculinizing and feminizing GAHT with twice-yearly subcutaneous lenacapavir (LEN) PrEP.

**Figure:**
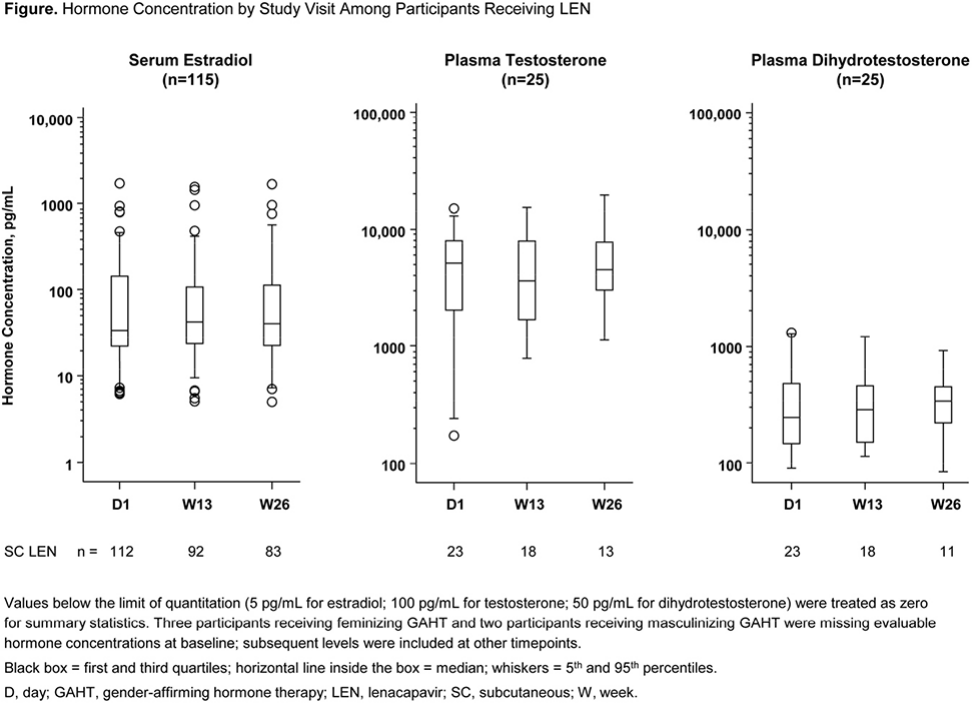

**Methods:**

PURPOSE 2 (NCT04925752), a Phase 3, randomized, controlled trial, demonstrated the efficacy of twice-yearly LEN PrEP in cisgender men, transgender women, transgender men, and gender nonbinary individuals. Participant characteristics, including self-reported gender identity, were collected at baseline. GAHT use was queried at each study visit. Plasma/serum concentrations of estradiol, testosterone, and dihydrotestosterone (testosterone active metabolite) were evaluated in a subset of gender-diverse participants receiving LEN and uninterrupted estradiol or testosterone for 26 weeks. Population PK (popPK) analyses assessed the impact of feminizing or masculinizing GAHT on LEN PK compared with LEN PK in participants not taking GAHT.

**Results:**

Among 2183 participants who received LEN in PURPOSE 2, 486 (22.3%) self-identified as gender diverse: 315 (14.4%) transgender women; 29 (1.3%) transgender men; 136 (6.2%) gender nonbinary; and 6 (0.3%) other gender. Concomitant GAHT use was reported by 253 (11.6%) participants. In a subset of participants assigned male at birth and taking estradiol (n=115) or assigned female at birth and taking testosterone (n=25), GAHT concentrations at weeks 13 and 26 were comparable with baseline (pre-dosing) levels, irrespective of dose, frequency, or any changes in dosing (Fig). PopPK analyses demonstrated that GAHT co-administration did not have a statistically significant impact on LEN PK.

**Conclusion:**

In the most gender-diverse Phase 3 PrEP trial conducted to date, LEN co-administration had no clinically significant impact on feminizing or masculinizing GAHT concentrations; nor did GAHT use impact LEN PK. These data support the co-administration of LEN and GAHT in gender-diverse individuals who are at disproportionately high likelihood of HIV acquisition.

**Disclosures:**

Jill Blumenthal, MD, MAS, Aspire Scientific: Medical writing support|Gilead Sciences, Inc.: Advisor/Consultant|Gilead Sciences, Inc.: Grant/Research Support|Gilead Sciences, Inc.: Medical writing support Maribel Acevedo-Quinones, MD, Aspire Scientific: Medical writing support|Gilead Sciences, Inc.: Medical writing support Nittaya Phanuphak, MD, PhD, Aspire Scientific: Medical writing support|Gilead Sciences, Inc.: Grant/Research Support|Gilead Sciences, Inc.: Medical writing support|ViiV Healthcare: Grant/Research Support Juan Carlos Hinojosa, MD, Aspire Scientific: Medical writing support|Gilead Sciences, Inc.: Medical writing support Javier R. Lama, MD, MPH, Aspire Scientific: Medical writing support|Gilead Sciences, Inc.: Grant/Research Support|Gilead Sciences, Inc.: Medical writing support Michelle S. Cespedes, MD, MS, Aspire Scientific: Medical writing support|Gilead Sciences, Inc.: Medical writing support|ViiV Healthcare: Advisor/Consultant Nkosiphile Ndlovu, MBChB, Dip. HIV Man, Aspire Scientific: Medical writing support|Gilead Sciences, Inc.: Medical writing support Carlos Brites, MD, PhD, Aspire Scientific: Medical writing support|Gilead Sciences, Inc.: Medical writing support|GSK: Board Member|GSK: Grant/Research Support Sarah B. Puryear, MD, MPH, Aspire Scientific: Medical writing support|Gilead Sciences, Inc.: Medical writing support and Employee|Gilead Sciences, Inc.: Stocks/Bonds (Public Company) Lillian B. Brown, MD, PhD, Aspire Scientific: Medical writing support|Gilead Sciences, Inc.: Medical writing support and Employee|Gilead Sciences, Inc.: Stocks/Bonds (Public Company) Christoph C. Carter, MD, PhD, Aspire Scientific: Medical writing support|Gilead Sciences, Inc.: Medical writing support and Employee|Gilead Sciences, Inc.: Stocks/Bonds (Public Company) Priyanka Arora, PhD, Aspire Scientific: Medical writing support|Gilead Sciences, Inc.: Medical writing support and Employee|Gilead Sciences, Inc.: Stocks/Bonds (Public Company) Marjorie Z. Imperial, PhD, Aspire Scientific: Medical writing support|Gilead Sciences, Inc.: Medical writing support and Employee|Gilead Sciences, Inc.: Stocks/Bonds (Public Company) Kim Etchevers, MS, Aspire Scientific: Medical writing support|Gilead Sciences, Inc.: Medical writing support and Employee|Gilead Sciences, Inc.: Stocks/Bonds (Public Company) Renu Singh, PhD, MS, Aspire Scientific: Medical writing support|Gilead Sciences, Inc.: 1475-US-PSP, 1475-WO-PCT, 1474-US-PSP, 1515-US-PSP, 1515-WO-PCT|Gilead Sciences, Inc.: Medical writing support and Employee|Gilead Sciences, Inc.: Stocks/Bonds (Public Company) Pamela Wong, MPH, Aspire Scientific: Medical writing support|Gilead Sciences, Inc.: Medical writing support and Employee|Gilead Sciences, Inc.: Stocks/Bonds (Public Company) Olivia T. Van Gerwen, MD, MPH, Abbott: Advisor/Consultant|Aspire Scientific: Medical writing support|BioNTech: Grant/Research Support|Gilead Sciences, Inc.: Grant/Research Support|Gilead Sciences, Inc.: Medical writing support|GSK: Advisor/Consultant|National Institute for Allergy and Infectious Diseases: Grant/Research Support

